# Liver resection for colorectal liver-limited metastases in elderly patients: a propensity score matching analysis

**DOI:** 10.1186/s12957-020-02055-8

**Published:** 2020-10-24

**Authors:** Ke-Min Jin, Kun Wang, Quan Bao, Hong-Wei Wang, Bao-Cai Xing

**Affiliations:** Key laboratory of Carcinogenesis and Translational Research (Ministry of Education/Beijing), Hepatobiliary and Pancreatic Surgery Unit I, Peking University Cancer Hospital & Institute, No. 52, Fu Cheng Road, Beijing, 100142 People’s Republic of China

**Keywords:** Liver-limited metastases, Colorectal cancer, Elderly, Hepatectomy, Survival

## Abstract

**Background:**

Few studies have focused on the role of hepatectomy for colorectal liver-limited metastases in elderly patients compared to matched younger patients.

**Methods:**

From January 2000 to December 2018, 724 patients underwent hepatectomy for colorectal liver-limited metastases. Based on a 1:2 propensity score matching (PSM) model, 64 elderly patients (≥ 70 years of age) were matched to 128 younger patients (< 70 years of age) to obtain two balanced groups with regard to demographic, therapeutic, and prognostic factors.

**Results:**

There were 73 elderly and 651 younger patients in the unmatched cohort. Compared with the younger group (YG), the elderly group (EG) had significantly higher proportion of American Society of Anesthesiologists score III and comorbidities and lower proportion of more than 3 liver metastases and postoperative chemotherapy (*p* < 0.05). After PSM for these factors, rat sarcoma virus proto-oncogene/B-Raf proto-oncogene (RAS/BRAF) mutation status and primary tumor sidedness, the EG had significantly less median intraoperative blood loss than the YG (175 ml vs. 200 ml, *p* = 0.046), a shorter median postoperative hospital stay (8 days vs. 11 days, *p* = 0.020), and a higher readmission rate (4.7% vs.0%, *p* = 0.036). The EG also had longer disease-free survival (DFS), overall survival (OS), and cancer-specific survival (CSS) compared to the YG, but these findings were not statistically significant (*p* > 0.05). Old age was not an independent factor for DFS, OS, and CSS by Cox multivariate regression analysis (*p* > 0.05).

**Conclusions:**

Hepatectomy is safe for colorectal liver-limited metastases in elderly patients, and these patients may subsequently benefit from prolonged DFS, OS, and CSS.

## Background

In 2018, colorectal cancer had the third greatest incidence and the second greatest mortality of all malignant tumors [1]. Approximately half of all colorectal cancer patients have disease that eventually progresses to synchronous or metachronous liver metastases (LM) with or without extrahepatic diseases (EHD) [2, 3]. Radical resection of all liver metastases is the mainstay of management for these patients, which has led to 5-year survival rates of 36 to 58% [4–9]. However, with the aging of the global population, an increasing number of patients are being diagnosed at an elderly age, when resection may not be a viable treatment option due to a patient’s poor performance status or comorbidities [10]. For the elderly patients who do undergo hepatectomy, there have been conflicting results of studies regarding operation safety and long-term survival [11–19]. These studies were not conclusive in part due to biased or missing baseline data, such as comorbidities, the American Society of Anesthesiologists (ASA) score, and preoperative treatment in these studies. To the best of our knowledge, only one study using the propensity score matching method has been published, which demonstrated comparable short-term and long-term outcomes between the younger group (YG) and the elderly group (EG) [20].

Another flaw of these studies, which investigated the justification for liver resection for colorectal liver metastases in elderly patients, is that they did include patients with EHD. Furthermore, most of the studies did not elaborate on the type and/or management of the EHDs, which could potentially hinder accurate evaluation of disease-free survival. In modern management of colorectal liver metastases, important data such as primary sidedness, RAS/BRAF mutation status, preoperative chemotherapy, and clinical risk score (CRS) were not included in the analyses. The present study was designed to elucidate the role of hepatectomy in elderly patients with colorectal liver-limited metastases using a propensity score matching analysis to overcome the aforementioned limitations of previous work.

## Methods

### Data collection

Data from patients with colorectal liver-limited metastases who underwent complete resection of hepatic metastases between January 2000 and December 2018 at the Hepatobiliary and Pancreatic Surgery Unit I of Beijing Cancer Hospital were retrospectively collected. The study was approved by the hospital’s Clinical Research Ethics Committee and was performed in compliance with the Helsinki Declaration. Patients with a second primary malignant tumor were excluded. The bowel and the hepatic resections were performed by a colorectal team and a hepaticobiliary team respectively. For patients with synchronous disease, liver resection was performed if the tumor could be safely resected after assessment of liver remnant reserve and performance status. For those patients with a heavy tumor burden in liver, especially after conversion therapy, liver first approach was adopted usually 2–4 weeks after cessation of preoperative chemotherapy. For those patients with rectal cancer, staged resection was preferred. Otherwise, simultaneous resection was performed. Only the first operation was included for patients who underwent a repeat hepatectomy for disease recurrence. For patients who underwent complete resection by two-stage hepatectomy (portal vein ligation or embolization), only the second surgery—the high-risk right hemi-liver resection—was included. For the purpose of liver parenchyma preservation, we tried to avoid major liver resection (≥ 3 segments) for patients with liver metastases. So, when the patient had tumors, which were smaller than 2 cm in diameter in the deep areas of liver and were not adjacent to large vessels or bile ducts, intraoperative RFA was utilized to reduce the parenchyma loss from otherwise liver resection. All tumor tissues resected prior to 2015 were retrieved and sent for retrospective RAS/BRAF mutation analysis. The patients were followed up every 6 months by hepaticobiliary team by outpatient clinic visit and telephone conversation. The following patient information was evaluated: (1) demographic features, comorbidities and ASA score; (2) primary tumor sidedness, T stage and N stage; (3) number, distribution, and maximum diameter of liver metastases; (4) preoperative serum levels of the tumor marker carcinoembryonic antigen (CEA) and carbohydrate antigen 19-9 (CA19-9), temporal relationship of primary tumor and liver metastases, preoperative CRS, preoperative chemotherapy, and RAS/BRAF mutation status; (5) operation time, procedural details (major hepatectomy, combination with radiofrequency ablation (RFA), simultaneous resection, Pringle clamping time of the hepaticoduodenal ligament), intraoperative blood loss, and red blood cell (RBC) transfusion and margin status; (6) postoperative hospital stay and postoperative general and surgical complications; (7) postoperative adjuvant chemotherapy protocol and history of repeat hepatectomy after recurrence; and (8) postoperative follow-up records of recurrence and death.

### Study population

All patients who underwent complete resection of colorectal liver-limited metastases with confirmed pathologic diagnosis were enrolled in this study. Seventy years of age was defined as the minimum age for elderly patients. Thus, the patients are divided into two groups—the YG (< 70 years of age) and the EG (≥ 70 years of age).

### Statistical analysis

Categorical variables were expressed as proportions and numerical variables were expressed as median and range. Categorical variables were compared by the chi-square or Fisher’s exact tests as appropriate whereas numerical variables were compared using the Mann-Whitney *U* test. The linear correlation coefficient was used to assess a potential relationship between two numerical variables. To compensate for the biases between the YG and the EG in the unmatched cohort, the propensity score (PS) “nearest neighbor” matching method was used with a matching ratio of 1:2 for the EG and the YG. The caliper value was set at 0.05. The standardized mean difference was used to assess the imbalance before and after PS matching. The following variables were included in the PS matching model: gender, ASA score, comorbidities, primary N stage, number of liver metastases, preoperative CEA, preoperative CRS score, RAS/BRAF mutation status, preoperative chemotherapy cycles, history of major liver resection, history of hepatectomy combined with intraoperative RFA, history of repeat hepatectomy after recurrence, and post-hepatectomy adjuvant chemotherapy. Response to preoperative chemotherapy was not included in the PS matching model due to too much missing data. Short-term results such as operation time, intraoperative blood loss, intraoperative and postoperative RBC transfusion, postoperative hospital stay, ICU stay, and Clavien-Dindo grades of general or surgical complications were compared between the EG and the YG before and after PS matching. Recurrence and survival data were followed up by telephone or outpatient visit every 6 months following hepatectomy. Disease-free survival (DFS) was defined as the duration from the date of hepatectomy to the date of event (tumor recurrence or death) or the last follow-up. Overall survival (OS) was defined as the duration from the date of hepatectomy to the date of death or the last follow-up. Cancer-specific survival (CSS) was defined as the duration from the date of hepatectomy to the date of cancer-related death or the last follow-up. Kaplan-Meier survival analysis was used to compare the DFS, OS, and CSS before and after PS matching by the log rank test. The Cox multivariate proportional hazards model was used to identify independent prognostic factors of DFS, OS, and CSS after PS matching. *P* < 0.05 was deemed significantly different. All statistical analyses were performed using an R-based extension bundle on the SPSS software package (version 23, SPSS Inc., Chicago, IL, USA).

## Results

### Comparison of elderly and younger patients before PS matching

#### Demographic characteristics and short-term outcomes

A total of 724 patients with colorectal liver-limited metastases were included in the study, with 73 patients in the EG and 651 patients in the YG. As shown in Tables 1 and 2, the median age was 56 years old for the YG and 73 years old for the EG (*p* < 0.001). There were 55 patients (8.4%) in the YG and 25 patients (34.2%) in the EG with an ASA score of III (*p* < 0.001). There were 274 patients (42.1%) in the YG and 50 patients (68.5%) in the EG with associated comorbidities (*p* < 0.001). With regard to specific comorbidities, there was a significantly higher percentage of patients with hypertension and ischemic heart disease in the EG than in the YG (*p* < 0.001 and *p* = 0.036, respectively). A significantly greater proportion of patients had more than 3 liver metastases (31.3% vs. 19.2%, *p* = 0.032) or received more than six cycles of chemotherapy (13.2% vs 4.1%, *p* = 0.025) in the YG compared to the EG. In addition, a greater proportion of patients received postoperative adjuvant chemotherapy in the YG compared to the EG (67.9% versus 54.8%, *p* = 0.024). With regard to intraoperative information, a higher proportion of patients in the YG received combined hepatectomy and RFA compared to the EG (13.1% vs. 4.1%, *p* = 0.017). Regarding postoperative outcomes, the Clavien-Dindo grades of both general and surgical complications did not significantly differ between groups, nor did in-hospital or 90-day mortality (*p* > 0.05). However, significantly greater proportions of patients in the EG were admitted to the ICU or readmitted to the hospital postoperatively compared to the YG (11.0% vs. 2.8%, *p* = 0.003 and 4.1% vs. 0.5%, *p* = 0.016, respectively).

**Table 1:** Demographics comparison of the elderly and younger patients before PS matching

Variable	Aged < 70 years (*n* = 651)	Aged ≥ 70 years (*n* = 73)	*P* value	Standardized mean difference
Age (years)	56 (19–69)	73 (70–83)	**< 0.001**	
Gender, male	417 (64.1%)	51 (69.9%)	0.325	0.173
ASA			**< 0.001**	0.487
I–II	596 (91.6%)	48 (65.8%)		
III–IV	55 (8.4%)	25 (34.2%)		
Comorbidity	274 (42.1%)	50 (68.5%)	**< 0.001**	0.564
Cerebrovascular disease	17 (2.6%)	5 (6.8%)	0.061	0.134
Arrhythmia	9 (1.4%)	1 (1.4%)	1.000	<0.001
Ischemic heart disease	36(5.5%)	9(12.3%)	**0.036**	0.474
Diabetes mellitus	94 (14.4%)	15 (20.5%)	0.166	0.265
Hypertension	178 (27.3%)	40 (54.8%)	**< 0.001**	0.648
Chronic obstructive pulmonary disease	5 (0.8%)	2 (2.7%)	0.151	0.362
Chronic renal dysfunction	4 (0.6%)	1 (1.4%)	0.413	0.242
Accompanying liver disease	29 (4.5%)	2 (2.7%)	0.760	0.198
Primary tumor sideness			0.198	
Right side	112 (17.2%)	17 (23.3%)		
Left side	539 (82.8%)	56 (76.7%)		
Primary T			0.659	
pT1-T2	64 (9.8%)	6 (8.2%)		
pT3-T4	587 (90.2%)	67 (91.8%)		
Primary N			0.869	0.087
pN0	199 (30.6%)	23 (31.5%)		
pN1-2	452 (69.4%)	50 (68.5%)		
Number of liver metastases(LM)			**0.032**	0.674
≤ 3	447 (68.7%)	59 (80.8%)		
> 3	204 (31.3%)	14 (19.2%)		
Distribution of LM			0.084	
Unilobar	341 (52.4%)	46 (63.0%)		
Bilobar	310 (47.6%)	27 (37.0%)		
Maximum diameter of LM			0.631	
≤ 5 cm	566 (86.9%)	62 (84.9%)		
> 5 cm	85 (13.1%)	11 (15.1%)		
Temporal relationship			0.796	
Synchronous	367 (56.4%)	40 (54.8%)		
Metachronous	284 (44.6%)	33 (45.2%)		
Preoperative chemotherpy cycles			**0.025**	0.743
≤ 6 cycles	565 (86.8%)	70 (95.9%)		
> 6 cycles	86 (13.2%)	3 (4.1%)		
Preoperative clinical risk score (CRS)			0.590	0.234
0–2	344 (52.8%)	41 (56.2%)		
3–5	307 (47.2%)	32 (43.8%)		
RAS/BRAF mutation	215(33.0%)	27(37.0%)	0.496	0.286
Preoperative CEA (ng/ml)	8.19 (0.47–1351.00)	8.53 (1.23–224.80)	0.566	0.344
Preoperative CA199(U/ml)	22.03 (0.00–29909.00)	24.56 (0.00–1354.00)	0.633	
Repeat resection after recurrence	73 (11.2%)	8 (11.0%)	0.948	0.063
Postoperative chemotherapy	442 (67.9%)	40 (54.8%)	**0.024**	0.766

**Table 2:** Comparison of intraoperative factors and postoperative short-term and long-term results before PS matching

Variable	Aged < 70 years (*n* = 651)	Aged ≥ 70 years (*n* = 73)	*P* value	Standardized mean difference
Combined with RFA	85 (13.1%)	3 (4.1%)	**0.027**	0.486
Two-stage hepatectomy	9 (1.4%)	1 (1.4%)	1.000	
Simultaneous resection	120 (18.4%)	17 (23.3%)	0.315	
Major hepatectomy	127 (19.5%)	9 (12.3%)	1.136	0.127
Pringle clamp	513 (78.8%)	53 (72.6%)	0.224	
Pringle clamp time (min)	20 (0–98)	15 (0–60)	0.061	
R1 margin	96 (14.7%)	7 (9.6%)	0.232	
Intraoperative blood loss (ml)	200 (0–6500)	150 (20–1000)	0.529	
Intraoperative RBC transfusion	28 (4.3%)	5 (6.8%)	0.367	
Intraoperative RBC transfused (U)	4 (1–12)	3 (2–4)	0.269	
Operation time (min)	187 (32–600)	180 (60–330)	0.146	
Hospital stay (days)	9 (4–78)	9 (4–48)	0.909	
Postoperative complications	186 (28.6%)	20 (27.4%)	0.833	
Clavien-Dindo classification			0.262	
I–II	106 (57.0%)	14 (70.0%)		
III–V	80 (43.0%)	6 (30.0%)		
General complications	58 (8.9%)	4 (5.5%)	0.321	
Postoperative heart failure	2 (0.3%)	0 (0%)	1.000	
Postoperative coronary artery disease	6 (0.9%)	0 (0%)	1.000	
Postoperative arrhythmia	8 (1.2%)	1 (1.4%)	1.000	
Postoperative lung infection	5 (0.8%)	1 (1.4%)	0.473	
Postoperative renal failure	2 (0.3%)	0 (0%)	1.000	
Postoperative pulmonary embolism	1 (0.2%)	0 (0%)	1.000	
Postoperative deep vein thrombosis	2 (0.3%)	0 (0%)	1.000	
Postoperative urinary infection	4 (0.6%)	0 (0%)	1.000	
Postoperative pleural effusion	28 (4.3%)	2 (2.7%)	0.759	
Postoperative stress ulcer	6 (0.9%)	1 (1.4%)	0.526	
Surgical complications	129 (19.8%)	11 (15.1%)	0.330	
Posthepatectomy liver failure	22 (3.4%)	2 (2.7%)	1.000	
Postoperative abdominal infection	53 (8.1%)	7 (9.6%)	0.671	
Postoperative bile leakage	37 (5.7%)	3 (4.1%)	0.788	
Postoperative abdominal collection	22 (3.4%)	2 (2.7%)	1.000	
Incision infection	9 (1.4%)	0 (0%)	0.610	
Postoperative ileus	12 (1.8%)	0 (0%)	0.622	
Postoperative abdominal bleeding	26 (4.0%)	1 (1.4%)	0.508	
ICU	18 (2.8%)	8 (11.0%)	**0.003**	
ICU stay (days)	1 (1–6)	1.5 (1–6)	0.927	
Postoperative RBC transfusion	50 (7.7%)	7 (9.6%)	0.566	
Postoperative RBC transfused (U)	4 (2–58)	2 (2–6)	0.510	
Reoperation	12 (1.8%)	0 (0%)	0.622	
Readmission	3 (0.5%)	3 (4.1%)	**0.016**	
Mortality (in-hospital)	1 (0.2%)	0 (0%)	1.000	
Mortality (90-day)	1 (0.2%)	0 (0%)	1.000	
Recurrence	467 (71.7%)	49 (67.1%)	0.409	
Intrahepatic recurrence	355 (54.5%)	40 (54.8%)	0.966	
Extrahepatic recurrence	112 (17.2%)	9 (12.3%)	0.290	
Disease-free survival			0.374	
1-year	46.6%	50.5%		
3-year	26.2%	31.0%		
5-year	23.5%	25.5%		
Overall survival			0.219	
1-year	94.1%	90.4%		
3-year	60.5%	56.3%		
5-year	48.7%	43.6%		
Cancer-specific survival			0.512	
1-year	94.4%	90.4%		
3-year	61.1%	59.0%		
5-year	49.2%	45.7%		

#### Long-term outcomes

**Fig. 1:**
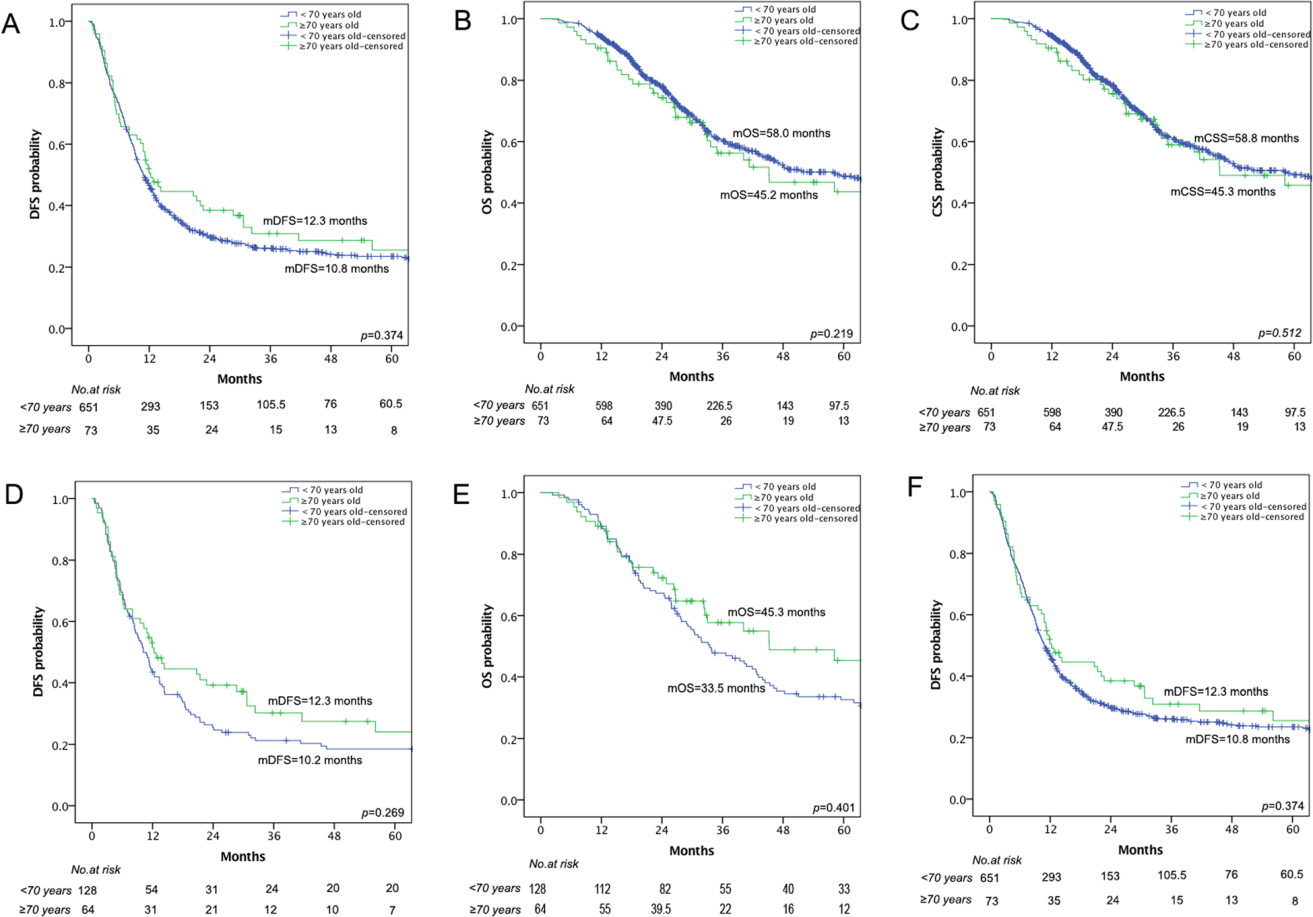
Comparison of disease-free survial (DFS), overall survival (OS), and cancer-specific survival (CSS) between the elderly group (EG) and the younger group (YG) before (**a**–**c**) and after propensity score matching (**d**–**f**)

The median follow-up period was 28.4 months. The recurrence rates—including both intrahepatic and extrahepatic recurrence—were not significantly different between the EG and YG (*p* > 0.05). There was also no significant difference in 1-year, 3-year, or 5-year DFS, OS, or CSS survival rates (*p* > 0.05; Table 2 and Fig. 1a–c).

### Comparison of elderly and younger patients after PS matching

#### Demographic characteristics and short-term outcomes

After PS matching for preoperative and prognostic factors that differed significantly between groups, a total of 64 cases from the EG and 128 cases from the YG were considered for matched analysis. As shown in Tables 3 and 4, the median ages of the EG and YG groups were 72.5 and 57 years old, respectively (*p* < 0.001). When the biases associated with differences in ASA score, comorbidities, number of liver metastases, number of preoperative chemotherapy cycles, intraoperative RFA, and postoperative adjuvant chemotherapy were removed by PS matching, the following intraoperative and postoperative differences between the groups were found. First, the median intraoperative blood loss in the YG was significantly higher than that in the EG (200 mL vs 175 mL, *p* = 0.046), and the median postoperative hospital stay was significantly longer in the YG than the EG (11 days vs. 8 days, *p* = 0.020). However, the readmission rate of the EG was still significantly greater than that of YG (4.7% vs. 0.0%, *p* = 0.036). Other postoperative variables, such as Clavien-Dindo grades for surgical and general complications, postoperative ICU stay, postoperative RBC transfusion, and reoperation rate, were not significantly different between groups (*p* > 0.05). In addition, the in-hospital and 90-day mortality rates were similar between the two groups (*p* > 0.05).

**Table 3:** Demographics comparison of the elderly and younger patients after PS matching

Variable	Aged < 70 years (*n* = 128)	Aged ≥ 70 years (*n* = 64)	*P* value	Standardized mean difference
Age (years)	57 (31–69)	72.5 (70–83)	**< 0.001**	
Gender, male	73 (57.0%)	43 (67.2%)	0.175	0.075
ASA			0.200	0.083
I–II	106 (82.8%)	48 (75.0%)		
III	22 (17.2%)	16 (25.0%)		
Comorbidity	65 (50.8%)	41 (61.1%)	0.081	0.086
Cerebrovascular disease	4 (3.1%)	2 (3.1%)	1.000	< 0.001
Arrhythmia	3 (2.3%)	1 (1.6%)	1.000	< 0.001
Ischemic heart disease	11 (8.6%)	6 (9.4%)	0.857	0.076
Diabetes mellitus	21 (16.4%)	12 (18.8%)	0.685	0.089
Hypertension	49 (38.3%)	31 (48.4%)	0.178	0.077
Chronic obstructive pulmonary disease	0 (0.0%)	1 (1.6%)	0.333	0.083
Chronic renal dysfunction	0 (0.0%)	1 (1.6%)	0.333	0.065
Accompanying liver disease	10 (7.8%)	2 (3.1%)	0.343	0.076
Primary tumor sideness			0.447	
Right side	24 (18.8%)	15 (23.4%)		
Left side	104 (81.3%)	49 (76.6%)		
Primary T			0.557	
pT1-T2	8 (6.3%)	6 (9.4%)		
pT3-T4	120 (93.8%)	58 (90.6%)		
Primary N			0.653	0.035
pN0	36 (28.1%)	20 (31.3%)		
pN1-2	92 (71.9%)	44 (68.8%)		
Number of liver metastases(LM)			0.433	0.056
≤ 3	106 (82.8%)	50 (78.1%)		
> 3	22 (17.2%)	14 (21.9%)		
Distribution of LM			1.000	
Unilobar	80 (62.5%)	40 (62.5%)		
Bilobar	48 (37.5%)	24 (37.5%)		
Maximum diameter of LM			0.509	
≤ 5 cm	103(80.5%)	54(84.4%)		
> 5 cm	25(19.5%)	10(15.6%)		
Temporal relationship			0.126	
Synchronous	61 (47.7%)	38 (59.4%)		
Metachronous	67 (52.3%)	26 (40.6%)		
Preoperative chemotherpy cycles			1.000	< 0.001
≤ 6 cycles	122 (95.3%)	61 (95.3%)		
> 6 cycles	6 (4.7%)	3 (4.7%)		
Preoperative clinical risk score (CRS)			0.535	0.065
0–2	76 (59.4%)	35 (54.7%)		
3–5	52 (40.6%)	29 (45.3%)		
RAS/BRAF mutation	47 (36.9%)	22 (34.4%)	0.750	0.046
Preoperative CEA (ng/ml)	9.10 (0.93–794.50)	9.41 (1.23–224.80)	0.858	0.102
Preoperative CA199 (U/ml)	28.11 (0.00–28,385.00)	24.06 (0.00–1354.00)	0.360	
Repeat resection after recurrence	20 (15.6%)	8 (12.5%)	0.563	0.078
Postoperative chemotherapy	86 (67.2%)	38 (59.4%)	0.286	0.094
Preoperative chemotherapy regimes			0.063	
Oxaliplatin-based	37 (28.9%)	29 (45.3%)		
Irinotecan-based	8 (6.3%)	5 (7.8%)		
5-Fu-based	1 (0.8%)	2 (3.1%)		
Hepatic artery infusion	4 (3.1%)	1 (1.6%)		
Postoperative chemotherapy regimes			0.601	
Oxaliplatin-based	50 (39.1%)	28 (43.8%)		
Irinotecan-based	22 (17.2%)	6 (9.4%)		
5-Fu-based	8 (6.3%)	3 (4.7%)		
Hepatic artery infusion	1 (0.8%)	0 (0%)

**Table 4:** Comparison of intraoperative factors and postoperative short-term and long-term results after PS matching

Variable	Aged < 70 years (*n* = 128)	Aged ≥ 70 years (*n* = 64)	*P* value	Standardized mean difference
Combined with RFA	1 (0.8%)	3 (4.7%)	0.109	0.043
Two-stage hepatectomy	1 (0.8%)	1 (1.6%)	1.000	
Simultaneous resection	17 (13.3%)	16 (25.0%)	0.066	
Major hepatectomy	24 (18.8%)	8 (12.5%)	0.273	0.023
Pringle clamp	95 (74.2%)	44 (68.8%)	0.424	
Pringle clamp time (min)	15 (0–60)	15 (0–60)	0.465	
R1 margin	3 (2.3%)	6 (9.4%)	0.062	
Intraoperative blood loss (ml)	200 (20–6500)	175 (20–800)	**0.046**	
Intraoperative RBC transfusion	10 (7.9%)	3 (4.7%)	0.549	
Intraoperative RBC transfused (U)	0 (0–8)	0 (0–4)	0.313	
Operation time (min)	172.5 (60–570)	180 (60–327)	0.799	
Hospital stay (days)	11 (4–70)	8 (4–48)	**0.020**	
Postoperative complications	37 (28.9%)	17 (26.6%)	0.733	
Clavien-Dindo classification			0.062	
I–II	16 (43.2%)	12 (70.6%)		
III–V	21 (56.8%)	5 (29.4%)		
General complications	15 (11.7%)	4 (6.3%)	0.232	
Postoperative heart failure	0 (0.0%)	0 (0%)	-	
Postoperative coronary artery disease	2 (1.6%)	0 (0%)	0.553	
Postoperative arrhythmia	0 (0.0%)	1 (1.6%)	0.333	
Postoperative lung infection	1 (0.8%)	1 (1.6%)	1.000	
Postoperative renal failure	0 (0.0%)	0 (0.0%)	-	
Postoperative pulmonary embolism	1 (0.8%)	0 (0.0%)	1.000	
Postoperative deep vein thrombosis	0 (0.0%)	0 (0.0%)	-	
Postoperative urinary infection	0 (0.0%)	0 (0.0%)	-	
Postoperative pleural effusion	10 (7.8%)	2 (3.1%)	0.343	
Postoperative stress ulcer	1 (0.8%)	1 (1.6%)	1.000	
Surgical complications	24 (18.8%)	8 (12.5%)	0.273	
Posthepatectomy liver failure	2 (1.6%)	1 (1.6%)	1.000	
Postoperative abdominal infection	3 (2.3%)	5 (7.8%)	0.120	
Postoperative bile leakage	8 (6.3%)	2 (3.1%)	0.500	
Postoperative abdominal collection	4 (3.1%)	2 (3.1%)	1.000	
Incision infection	3 (2.3%)	0 (0.0%)	0.552	
Postoperative ileus	4 (3.1%)	0 (0.0%)	0.303	
Postoperative abdominal bleeding	5 (3.9%)	1 (1.6%)	0.666	
ICU	5 (3.9%)	6 (9.4%)	0.185	
ICU stay (days)	2 (1–6)	1.5 (1–6)	0.537	
Postoperative RBC transfusion	11 (8.6%)	7 (10.9%)	0.599	
Postoperative RBC transfused (U)	4 (2–58)	2 (2–6)	0.151	
Reoperation	4 (3.1%)	0 (0.0%)	0.303	
Readmission	0 (0.0%)	3 (4.7%)	**0.036**	
Mortality (in-hospital)	1 (0.8%)	0 (0.0%)	1.000	
Mortality (90-day)	1 (0.8%)	0 (0.0%)	1.000	
Recurrence	102 (79.7%)	43 (67.2%)	0.058	
Intrahepatic recurrence	70 (54.7%)	34 (53.1%)	0.838	
Extrahepatic recurrence	32 (25.0%)	9 (14.1%)	0.081	
Disease-free survival			0.269	
1-year	43.5%	51.4%		
3-year	21.2%	30.3%		
5-year	18.5%	24.1%		
Overall survival			0.401	
1-year	89.0%	89.1%		
3-year	47.8%	57.7%		
5-year	32.7%	45.4%		
Cancer-specific survival			0.163	
1-year	89.8%	89.1%		
3-year	48.2%	61.1%		
5-year	32.9%	48.0%

**Table 5:** Multivariate Cox regression analyses of disease-free survival, overall survival, and cancer-specific survival after PS matching

Variable	Relative ratio	95% Confidence interval	*P* value
Disease-free survival			
Age (≥ 70 years old)	0.860	0.602–1.230	0.409
RAS/BRAF mutation	1.558	1.117–2.174	**0.009**
Preoperative CEA (≥ 20 ng/ml)	1.635	1.155–2.314	**0.006**
Preoperative CRS (≥ 3)	1.637	1.142–2.347	**0.007**
Number of Liver metastases (> 3)	1.732	1.120–2.678	**0.014**
Overall survival			
Age (≥ 70 years old)	0.828	0.550–1.247	0.367
RAS/BRAF mutation	1.432	0.985–2.081	0.060
Preoperative CEA (≥ 20 ng/ml)	1.699	1.157–2.497	**0.007**
Preoperative CRS (≥ 3)	1.638	1.141–2.352	**0.007**
Cancer-specific survival			
Age (≥ 70 years old)	0.699	0.455–1.076	0.103
RAS/BRAF mutation	1.348	0.911–1.994	0.136
Preoperative CEA (≥ 20 ng/ml)	2.008	1.358–2.968	**< 0.001**
Preoperative CRS (≥ 3)	1.548	1.069–2.241	**0.021**

#### Long-term outcomes

The median follow-up period for the matched patient groups was 29.8 months. The recurrence rates—including both intra- and extra-hepatic recurrence—between the EG and YG were not significantly different (*p* > 0.05). The 1-year, 3-year, and 5-year DFS, OS, and CSS survival rates were higher in the EG than in the YG; however, this difference was not statistically significant (*p* > 0.05; Table 4 and Fig. 1d–f). In the EG, the 5-year DFS, OS, and CSS rates were 24.1%, 45.4%, and 48.0%, respectively; the median lengths of DFS, OS, and CSS were 12.3 months, 45.3 months, and 58.2 months respectively. In the YG, the 5-year DFS, OS, and CSS rates were 18.5%, 32.7%, and 32.9% respectively; the median lengths of DFS, OS, and CSS were 10.2 months, 33.5 months, and 33.5 months respectively.

#### Cox proportional hazards model analysis

Cox multivariate regression analysis was performed for the PS-matched cohort, which included 64 EG patients and 128 YG patients. As shown in Table 5, RAS/BRAF mutation status, preoperative serum CEA levels ≥ 20 ng/mL, preoperative CRS ≥ 3, and the presence of > 3 liver metastases were identified as independent predictive factors of DFS (*p* < 0.05). Preoperative serum CEA levels ≥ 20 ng/mL and preoperative CRS ≥ 3 were identified as independent predictive factors of both OS and CSS (*p* < 0.05). Notably, old age (≥ 70 years) was not identified as an independent predictive factor for DFS, OS, or CSS (*p* > 0.05).

## Discussion

With the increasing age of the global population, stage IV colorectal cancer is being diagnosed in elderly patients more frequently than ever before [10]. As a result of extensive progress in surgical and anesthetic techniques and modern chemotherapy regimens, more patients can undergo resection of liver metastases with curative intent. This has been proven to be the most effective treatment strategy for colorectal liver metastases (CRLM). However, the increasing possibility of age-related comorbidities and high ASA scores among the elderly patients undergoing hepatectomy poses a higher risk of postoperative morbidity and mortality. Most recently, a multidisciplinary consensus from Italy pointed out the potentially negative impact of old age on recovery of patients from damage. The consensus also put forward some useful evaluation benchmarks for the elderly before operation, which would be critical for the selection of the elderly patients for surgery in the futur e[21]. Although some previous literature reported that older patients had similar surgical safety and long-term survival compared with younger patients, baseline data for both groups were not balanced. Some important prognostic factors such as RAS/BRAF mutation and primary tumor sidedness were also not included in published studies. Therefore, this study, which compared the short-term and long-term results of hepatectomy for younger and elderly patients, was designed to overcome the abovementioned drawbacks.

There are different definitions in the literature as to the cutoff age for an individual to be designated as elderly [11, 15–18, 20]. However, the most frequently used cutoff age is 70 [15, 16, 22–24], which was adopted for the EG cutoff in this study. Due to the unmatched demographic and preoperative treatment data between the EG and the YG in this study, a PSM method was used to probe the effect of age on patient outcomes after hepatectomy. De Blasi et al. [20] also used this method to mitigate potential biases between groups. However, despite the PSM approach, there were still some unmatched parameters between the groups—namely pedicle clamping duration and recurrence treatment protocol—which resulted in some inconclusive analyses. Furthermore, most previous studies have enrolled patients with EHD, which makes accurate definition of DFS challenging. To overcome this disadvantage, we excluded all patients with EHD from analysis in this study.

Before propensity score matching, there were biases in the baseline data between the EG and the YG due to differences in comorbidities and ASA scores. In addition, some perioperative factors—such as the proportion of patients who had > 3 liver metastases, received more than six cycles of preoperative chemotherapy, received postoperative chemotherapy, or underwent intraoperative RFA—were also significantly different between the YG and EG. Importantly, it has been reported that more than six cycles of preoperative chemotherapy and intraoperative RFA may increase postoperative morbidity; furthermore, having > 3 liver metastases and receiving postoperative adjuvant chemotherapy are important prognostic factors for patients with CRLM [25, 26]. Propensity score matching was used to balance the distribution of these variables between groups in this study. After matching, short-term patient outcomes revealed that the EG had significantly less intraoperative blood loss and a shorter postoperative hospital stay—though a higher readmission rate—compared to the YG. Long-term outcomes demonstrate a slight, but non-statistically significant improvement, in 5-year DFS, OS, and CSS for the EG compared with the YG.

For modern treatment of CRLM, routine testing of RAS/BRAF mutation status has been recommended since 2014 as it has been confirmed to be a negative prognostic factor for CRLM patients [27]. It has been reported that positive RAS/BRAF mutation status is associated with shorter DFS and OS and narrower margin widths after hepatectomy compared to wild-type RAS/BRAF [28–30]. However, RAS/BRAF mutation status has never been considered in previous retrospective studies, as much of the data precedes standard testing for RAS/BRAF status. In this study, all tumor tissue samples from the considered patients were retrieved from the pathology department and tested for RAS/BRAF mutation status, which was matched between the two groups after PSM.

Primary tumor sidedness has also been emphasized in recent years in recognition of the fact that the side of origin plays a role in tumor behavior and progression. It was reported that tumors originating on the right were more frequently associated with female patients, the elderly, high grade (poor differentiation), BRAF mutations, the enhanced CpG island methylator phenotype, high microsatellite instability, and high expression of consensus molecular subtypes 1 and 3 compared with left-side origin tumors [31]. These characteristics negatively affect anti-EGFR treatment and the prognosis of patients with right-sided tumors [32]. Therefore, we included primary tumor sidedness in our PSM model to balance the possible bias associated with this disease feature.

The CRS was proposed in 1999 by Fong et al. [33] as a prognostic indicator composed of five preoperative variables: preoperative CEA > 200 ng/mL, primary positive lymph nodes, an interval of <12 months between diagnosis of the primary tumor and liver metastasis, presence of multiple liver metastases, and maximal diameter of liver metastases > 5 cm. It has been shown that increased CRS is associated with an increased risk of postoperative recurrence and death [34]. Thus, it was important to include CRS in our PSM analysis to appropriately examine the effect of age on survival.

This study revealed no significant difference in either general or surgical postoperative complications between the EG and the YG. This result aligns with previous studies and suggests that surgery for elderly patients is as safe as it would be for younger patients with the same ASA score and comorbidities [20, 35]. This holds even when simultaneous resection of primary and liver tumors or major hepatectomy is performed. Interestingly, compared to the YG, the EG was found to have significantly less intraoperative blood loss (175 mL vs. 200 mL, *p* = 0.046) and a shorter median postoperative hospital stay (8 days vs. 11 days, *p* = 0.020), which might reflect that appropriately selected elderly recover promptly from surgery. However, the EG group did have a significantly higher readmission rate than the YG (4.7% vs. 0%, *p* = 0.036). Although the median intraoperative blood loss of the YG is higher than that of the EG, an absolute difference of 25 mL is of little clinical significance. The significance of the difference between groups may result from the relatively small sample size of this study, considering the *p* value approaching 0.05. With a larger sample size and improved matching between the groups with regard to perioperative details—such as preoperative chemotherapy, major hepatectomy, simultaneous resection, and Pringle clamping time—the intraoperative blood loss will likely be comparable between the EG and the YG.

The three patients from the EG who were readmitted to the hospital were all 71 years of age. The first patient was diagnosed with synchronous descending colon cancer with liver metastases and received simultaneous resection of the primary tumor and liver metastases. The patient was readmitted due to incisional infection 2 months after discharge. The second readmitted patient was diagnosed with bilobar liver metastases after resection of sigmoid colon cancer. He was readmitted to the hospital 1 month after liver resection due to a fever of 38.3 °C. Laboratory tests showed a normal white blood cell count, and radiological examinations revealed no signs of abdominal or thoracic collection. The third readmitted patient was diagnosed with multiple liver metastases after resection of ascending colon cancer. He received hepatectomy and intraoperative RFA for multiple tumors. He was readmitted to the hospital 2 months after operation due to fever of 38.5 °C. The white blood cell count was marginally elevated, and ultrasound showed a small thoracic collection without abdominal fluid. In brief, although three patients from the EG were readmitted to the hospital, only one of them experienced an unequivocal surgical complication (incisional infection), which classified as a minor complication. None of the readmitted patients experienced systemic complications related to old age. When the length of hospital stay after readmission was added to the length of the postoperative hospital stay, the median total length of postoperative hospital stay was still significantly longer in the YG than in the EG (11 days vs. 8 days, *p* = 0.024). As such, we do not believe that the EG’s length of stay advantage compared to the YG was offset by the higher readmission rate.

With regard to the median length of postoperative stay being shorter in EG patients compared to YG patients, we found that the proportions of major hepatectomy and grade III or higher postoperative complications were much higher in the YG than the EG (18.8% vs. 12.5% and 56.8% vs. 29.4%, respectively), although this was not statistically significant. Given the relatively small sample size of our study, the significantly shorter postoperative length of stay in the EG may result from these differences. Thus, this result should be clarified in larger studies in the future. Regarding long-term patient outcomes, surprisingly, the 5-year DFS, OS, and CSS rates were increased by 5.6%, 12.7%, and 15.1%, respectively, for the EG compared to the YG, although these differences were not statistically significant. Importantly, it was shown that the OS and CSS of the YG were longer than those of the EG prior to PSM. This discrepancy may arise due to the following factors. First, some research [36, 37] has shown that the malignancy of tumors in the elderly population may be reduced; thus, the potential for tumor growth and metastasis may be decreased in the elderly as well. Second, some factors such as RAS/BRAF mutation status, primary tumor sidedness, and history of preoperative chemotherapy were included in our study and balanced between the EG and YG by PSM. These important prognostic factors were lacking in previously published studies, which may contribute to the difference in OS identified in our study. Notably, due to the relatively small sample size of this study, this conclusion should be confirmed by high-quality studies with a larger sample size in the future.

This study has some limitations. First, similar to other studies, this study had a relatively small sample size. After eliminating patients with EHD and unmatched patients, there were only 64 patients in the EG and 128 patients in the matched YG considered for analysis, which may impact the representativeness and robustness of the results. As such, the results of this study should be confirmed by high-quality studies with larger sample sizes in the future. Another limitation is the retrospective nature of this study, so the conclusion should be evaluated by large prospective controlled trials in future work. The third limitation is the loss of information regarding the response to preoperative chemotherapy as a referral center, which has been shown in previous work to be a pivotal prognostic factor for CRLM patients undergoing hepatectomy [34, 38]. Finally, the fourth limitation was that the chemotherapy regimes had changed during so long study interval. Actually, preoperative and postoperative chemotherapy backbone drugs of the younger group were not significantly different from those of the elderly group as shown in Table 3. However, the detailed chemotherapy regimes were very difficult to be balanced between the two groups.

## Conclusions

For appropriately identified elderly patients with colorectal liver-limited metastases, hepatectomy is safe and effective. In this study, we found that there was no increase in postoperative morbidities and mortality compared with matched younger patients. Importantly, elderly patients may benefit from longer DFS, OS, and CSS after hepatectomy; thus, this procedure should be performed for elderly patients who are in the same performance status with adequate cardiopulmonary reserve as the younger patients, especially for those patients with favorable biological behavior.

## Data Availability

The data that support the findings of this study are available on request from the corresponding author. The data are not publicly available due to privacy or ethical restrictions.

## Abbreviations

PSM: Propensity score matching
YG: Younger group
EG: Elderly group
OS: Overall survival
DFS: Disease-free survival
CSS: Cancer-specific survival
RAS: Rat sarcoma virus proto-oncogene
BRAF: B-Raf proto-oncogene
EHD: Extrahepatic diseases
ASA: American Society of Anesthesiologists
CRS: Clinical risk score
CEA: Carcinoembryonic antigen
CA19-9: Carbohydrate antigen 19-9
RBC: Red blood cell
RFA: Radiofrequency ablation
CRLM: Colorectal liver metastases
